# Supplementary material for: The Prodigious Potential of mRNA Electrotransfer as a Substitute to Conventional DNA-Based Transient Transfection
Source: Cells. 2023 Jun 8;12(12):1591. doi: 10.3390/cells12121591 (PMC10297312; doi:10.3390/cells12121591)
Supplement: Supplementary file 1 [file cells-12-01591-s001.zip › cells-2407390-supplementary.pdf]

Supporting Information

The Prodigious Potential of mRNA Electrotransfer as a Substitute to Conventional DNA-Based Transient Transfection

Théo Juncker, Bruno Chatton and Mariel Donzeau *

mariel.donzeau@unistra.fr

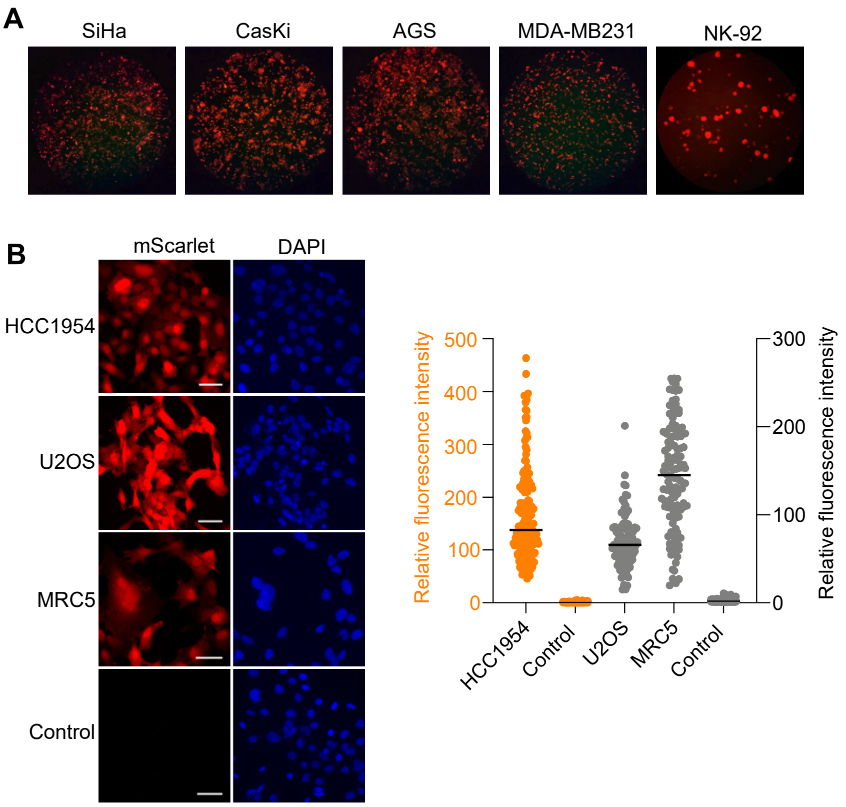

Figure S1. Transfection of different cell-lines with mRNA encoding for mScarlet using electroporation. (A) Living cells were visualized by direct fluorescence on inverted fluorescence microscope and images were taken by moticam 4000 full HD camera. (B) Cells were fixed, visualized by direct fluorescence and analyzed by ImageJ. 5.0. Bar scale 40 µm. Quantificiation was performed using ImageJ.

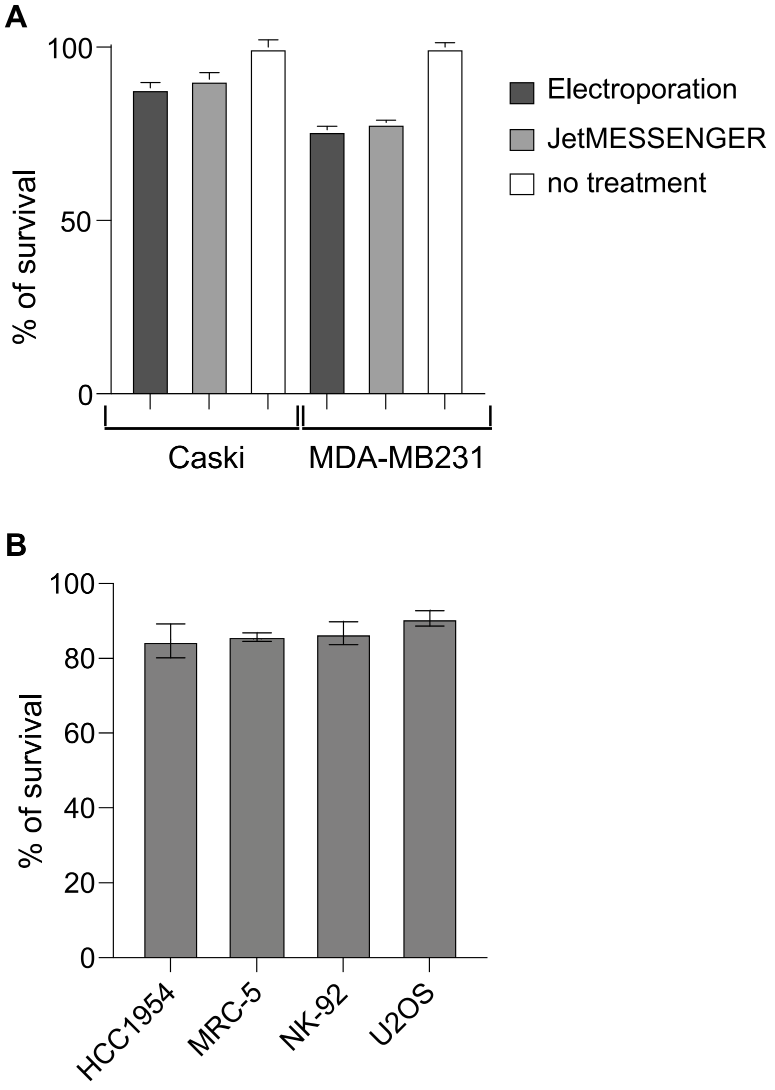

Figure S2. Survival studies. (A) Percentage of survival of Caski and MDA-MB231 after mScarlet coding mRNA transfection either using JetMESSENGER of electroporation compare to no treated cells. (B) Survival of HCC1954, MRC5, NK92 and U2OS following electroporation of mScarlet coding mRNA was assessed as % of non-transfected cells. (n≥ 200).

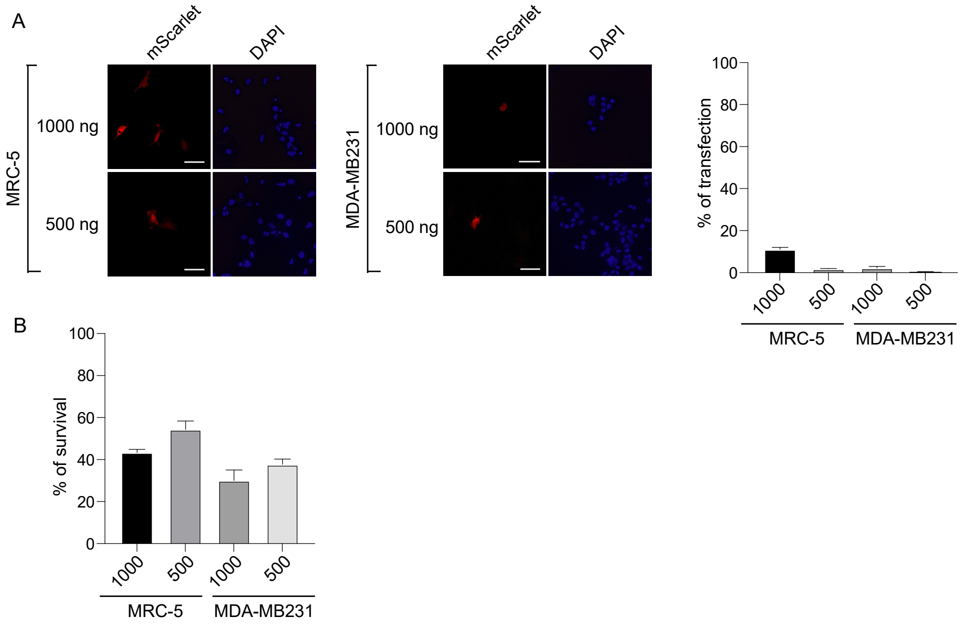

Figure S3. DNA transient transfection efficiency, and cell survival. (A) MRC-5 and MDA-MB213 were transfected with DNA coding for the mScarlet using electroporation and cells were analyzed by direct fluorescence (red). Nuclei were stained with DAPI (blue). Scale bar, 50 µm. Percentage of transfection efficacy was quantified by ImageJ and blotted (n≥100). (B) Survival was assessed as a % of cells compared to the non-transfected cells.

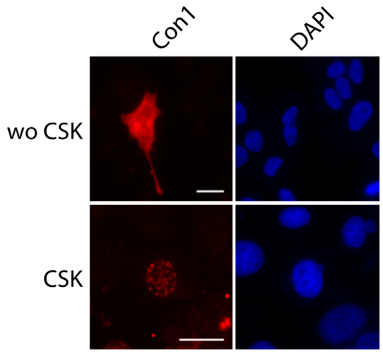

Figure S4. Cells were transfected with pβ-con1-mCherry expression plasmid using pEI. Cells were treated or not with CSK buffer before fixation. Cells were visualized by direct fluorescence and analyzed by ImageJ. Bar scale 20 µm.
